# The P-body component USP52/PAN2 is a novel regulator of *HIF1A* mRNA stability

**DOI:** 10.1042/BJ20130026

**Published:** 2013-03-28

**Authors:** John S. Bett, Adel F. M. Ibrahim, Amit K. Garg, Van Kelly, Patrick Pedrioli, Sonia Rocha, Ronald T. Hay

**Affiliations:** *Scottish Institute for Cell Signalling, Sir James Black Centre, College of Life Sciences, University of Dundee, Dow Street, Dundee DD1 5EH, Scotland, U.K.; †Wellcome Trust Centre for Gene Regulation and Expression, College of Life Sciences, University of Dundee, Dundee DD1 5EH, Scotland, U.K.

**Keywords:** AU-rich element (ARE)-mediated degradation (AMD), hypoxia-inducible factor 1α (HIF1A), poly(A) nuclease 2 (PAN2), processing body (P-body), pseudo-deubiquitinating enzyme (pseudo-DUB), ubiquitin-specific protease 52 (USP52), aHIF, antisense hypoxia-inducible factor, ARE, AU-rich element, AMD, ARE-mediated degradation, CA9, carbonic anhydrase IX, CHX, cycloheximide, CTNNB1, β-catenin, CUL2, cullin 2, DCP1A, decapping enzyme 1A, DUB, deubiquitinating enzyme, ERG, Ets-related gene, FBS, fetal bovine serum, FISH, fluorescent *in situ* hybridization, GFP, green fluorescent protein, GLUT1, glucose transporter 1, HEK, human embryonic kidney, HIF1A, hypoxia-inducible factor 1α, HIF1B, hypoxia-inducible factor 1β, HRE, hypoxia-response element, LC, liquid chromatography, LDHA, lactate dehydrogenase A, miRNA, microRNA, MS/MS, tandem MS, NEDD8, neural-precursor-cell-expressed developmentally down-regulated 8, NP-40, Nonidet P40, NT, Non-Targeting, PABPC1, poly(A)-binding protein C1, PAN2, poly(A) nuclease 2, P-body, processing body, PHD, prolyl hydroxylase, RT, reverse transcription, siRNA, short interfering RNA, TCE, transcription elongation factor, TRIM21, tripartite motif-containing 21, TTP, tristetrapolin, USP52, ubiquitin-specific protease 52, UTR, untranslated region, VEGF, vascular endothelial growth factor, VHL, von Hippel–Lindau, YFP, yellow fluorescent protein

## Abstract

HIF1A (hypoxia-inducible factor 1α) is the master regulator of the cellular response to hypoxia and is implicated in cancer progression. Whereas the regulation of HIF1A protein in response to oxygen is well characterized, less is known about the fate of *HIF1A* mRNA. In the present study, we have identified the pseudo-DUB (deubiquitinating enzyme)/deadenylase USP52 (ubiquitin-specific protease 52)/PAN2 [poly(A) nuclease 2] as an important regulator of the HIF1A-mediated hypoxic response. Depletion of USP52 reduced HIF1A mRNA and protein levels and resulted in reduced expression of HIF1A-regulated hypoxic targets due to a 3′-UTR (untranslated region)-dependent poly(A)-tail-length-independent destabilization in *HIF1A* mRNA. MS analysis revealed an association of USP52 with several P-body (processing body) components and we confirmed further that USP52 protein and *HIF1A* mRNA co-localized with cytoplasmic P-bodies. Importantly, P-body dispersal by knockdown of *GW182* or *LSM1* resulted in a reduction of *HIF1A* mRNA levels. These data uncover a novel role for P-bodies in regulating *HIF1A* mRNA stability, and demonstrate that USP52 is a key component of P-bodies required to prevent *HIF1A* mRNA degradation.

## INTRODUCTION

Cells respond to reduced oxygen tension by executing a transcriptional programme that is principally orchestrated by HIF1A (hypoxia-inducible factor 1α) [1]. HIF1A protein is synthesized continually, but degraded rapidly by the ubiquitin–proteasome system under normal oxygen concentrations (normoxia) [2,3]. This is a result of oxygen-dependent proline hydroxylation mediated by a family of PHDs (prolyl hydroxylases). HIF1A containing this hydroxyproline modification is a substrate for the VHL (von Hippel–Lindau) E3 ubiquitin ligase complex which targets the protein for ubiquitin-mediated proteolysis [4,5]. Upon decreased oxygen concentration (hypoxia), such as that observed in solid tumours, HIF1A escapes proline hydroxylation and degradation to bind its constitutively expressed partner HIF1B (hypoxia-inducible factor 1β) and drive the expression of many genes involved in glycolysis, angiogenesis, cell survival and cancer progression [3].

Whereas the regulation of HIF1A protein is well documented, little is known about the regulation and turnover of *HIF1A* mRNA. The presence of multiple AREs (AU-rich elements) in the 3′-UTR (untranslated region) of *HIF1A* and the observation that HuR binds this 3′-UTR suggested regulation of the *HIF1A* transcript via AMD (ARE-mediated degradation) [6]. In support of this, the presence of AREs in the HIF1A 3′-UTR has been reported to be necessary for TTP (tristetrapolin)-mediated degradation of *HIF1A* mRNA during prolonged hypoxia [7,8]. In addition, the existence of an aHIF (antisense hypoxia-inducible factor) complementary to 1027 bases in the *HIF1A* 3′-UTR has led to the proposal that *HIF1A* mRNA is targeted for degradation by binding of aHIF to its 3′-UTR and exposing AREs to TTP [9]. Indeed, aHIF was shown to be up-regulated by prolonged hypoxia and correlated with a reduction in *HIF1A* mRNA stability [10].

USP52 (ubiquitin-specific protease 52)/PAN2 [poly(A) nuclease 2] belongs to the ubiquitin-specific protease superfamily, but exhibits no deubiquitylating activity owing to the lack of an active-site cysteine residue [11]. It also contains a C-terminal exonuclease III domain and has been well characterized in its role as a poly(A) nuclease as part of the PAN2–PAN3 deadenylation complex [12,13]. PABPC1 [poly(A)-binding protein C1] recruits the PAN2–PAN3 complex to poly(A) tails through binding PAN3 and stimulating USP52/PAN2 poly(A) nuclease activity [14,15]. However, whereas *Saccharomyces cerevisiae* USP52/PAN2 deletion mutants accumulate longer poly(A) tails [12], they are viable because of the CCR4–NOT1 complex providing the major cellular deadenylation activity [16], and this is also similarly the case in mammalian cells [17]. USP52/PAN2 and PAN3 have also been reported to be components of cytoplasmic P-bodies (processing bodies) [18]. Interestingly, as PABPC1 is not present in P-bodies, but is required for USP52/PAN2 nuclease activity, it is likely that USP52/PAN2 has additional functions within P-bodies [18].

In the present study, screening has identified USP52/PAN2 as an important regulator of the HIF1A-mediated hypoxic response. USP52 was required for *HIF1A* mRNA stability, and we provide evidence that this acts through *HIF1A*'s AU-rich 3′-UTR, but is independent of poly(A) tail length regulation. Disrupting P-bodies by GW182 depletion displaces USP52 from P-bodies and reduces *HIF1A* mRNA levels. These data thereby reveal USP52 as a key component of P-bodies required for *HIF1A* mRNA stability.

## EXPERIMENTAL

### Cell culture, stable cell generation and DNA transfections

U2OS, HeLa, HEK (human embryonic kidney)-293 and RCC4 cells were maintained in DMEM (Dulbecco's modified Eagle's medium) (Gibco) supplemented with 10% (v/v) FBS (fetal bovine serum). 786-O cells were maintained in RPMI 1640 medium (Gibco) supplemented with 10% (v/v) FBS. U2OS-HRE cells [19] were maintained in 0.5 μg/ml puromycin (Sigma). U2OS-HRE cells stably expressing YFP (yellow fluorescent protein)–USP52 were generated by transfecting U2OS-HRE cells with pEFIRES-B-eYFP-USP52 and selecting with 10 μg/ml blasticidin 48 h after transfection then were pooled and maintained with 10 μg/ml blasticidin and 0.5 μg/ml puromycin. Tetracycline-inducible FLAG–USP52 HEK-293 cells were generated using the T-REx system (Invitrogen) according to the manufacturer's instructions and maintained in the presence of 5 μg/ml blasticidin and 100 μg/ml hygromycin. Induction was carried out using 1 μg/ml tetracycline. MLN4924 was used at 3 μM final concentration for 3 h and MG132 treatment was at 20 μM for 4 h. CHX (cycloheximide) (Sigma) treatment was performed for 2 h at 5 μg/ml and puromycin treatment was for 1 h at 100 μg/ml to modify P-bodies.

### Hypoxia treatment

Hypoxia experiments were performed by placing cells under 1% oxygen in an Invivo_2_ 300 workstation (Ruskinn) for 24 h. Cell extracts for protein and RNA were taken inside the workstation to avoid reoxygenation.

### Luciferase assays

Luciferase assays were performed by lysing cells in luciferase buffer [25 mM Tris/phosphate (pH 7.8), 8 mM MgCl_2_, 1 mM DTT (dithiothreitol), 1% (w/v) Triton X-100, 15% (v/v) glycerol, 0.5 mM ATP, 0.5% BSA, 0.125 mM luciferin and 4 μM sodium pyrophosphate] and reading counts on an Envision 2104 plate reader (PerkinElmer). Dual-luciferase assays were carried out on HEK-293 and U2OS cells co-transfected with either *Renilla* luciferase–*HIF1A*-3′-UTR or *Renilla* luciferase–*HIF2A*-3′-UTR and pcDNA3.1+-firefly luciferase using the Dual Luciferase Reporter assay system according to the manufacturer's instructions (Promega). *Renilla* luciferase counts were normalized to firefly luciferase to control for transfection efficiency. All luciferase assays were carried out in triplicate and represent at least two independent experiments.

### siRNA (short interfering RNA)

ON-TARGETplus Non-Targeting (NT), *USP52*, *GW182*, *LSM1* and *PAN3* (Dharmacon) siRNAs were used at a final concentration of 20 nM. siRNA transfections were carried out using Lipofectamine™ RNAi Max (Invitrogen) according to the manufacturer's instructions. *USP52* and *LSM1* siRNA treatments were for 48 h, *GW182* siRNA treatment was for 72 h and *PAN3* treatment was for 96 h. Sequences of individual siRNA duplexes are given in Supplementary Table S1 at http://www.biochemj.org/bj/451/bj4510185add.htm.

### Antibodies and Western blotting

**Figure 1:**
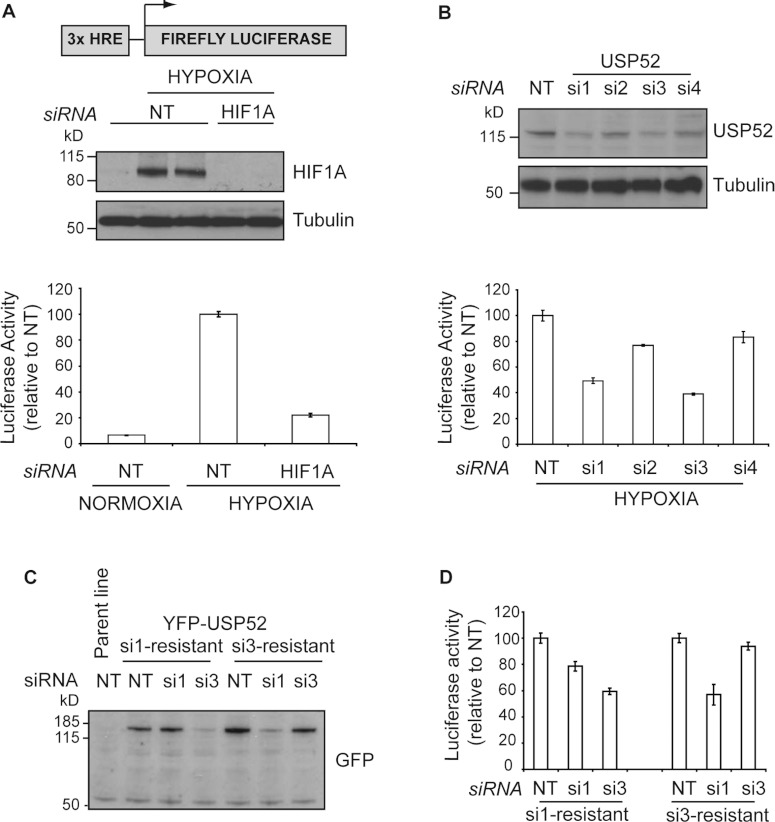
USP52 is a regulator of the hypoxia response (**A**) U2OS osteosarcoma cells stably expressing a hypoxia reporter construct consisting of three tandem HREs fused to the firefly luciferase gene (U2OS-HRE) were used to identify novel mediators of the hypoxia response. Western blot analysis demonstrates that HIF1A protein expression increases concomitant with a 10-fold increase in luciferase activity upon hypoxia treatment. Tubulin was used as a loading control. (**B**) Deconvolution analysis of USP52 revealed that individual siRNAs si1 and si3 both elicited an impaired response to hypoxia that correlated with the ability of these oligonucleotides to reduce USP52 protein levels by approximately 50%. Tubulin was used as a loading control. (**C**) Western blot of U2OS-HRE cells stably expressing siRNA-resistant YFP–USP52 were resistant to the individual siRNA as indicated, but remained sensitive to the other siRNA. (**D**) Luciferase assays of siRNA-resistant cells lines revealed USP52 si1 treatment was rescued to 80%, whereas si3 treatment was rescued to 95%. Cell lines remained sensitive to the individual siRNA to which resistance was not designed. Molecular masses are indicated in kDa in the blots, and results in histograms are means±S.E.M.

Cells were lysed in NP-40 (Nonidet P40) buffer [50 mM Hepes/KOH (pH 7.2), 400 mM NaCl, 1% NP-40, 0.2 mM EDTA and 10% (v/v) glycerol with protease inhibitor cocktail (Sigma)] and Western blotting was carried out using the following antibodies: anti-HIF1A (R&D Systems #MAB1536, mouse monoclonal, 1:1000 dilution) used in Figure 1(A) then anti-HIF1A (Novus #NB100-134, rabbit polyclonal, 1:1000 dilution) was used in all subsequent experiments, anti-tubulin (Sigma #T0198, mouse monoclonal, 1:2000 dilution), anti-USP52 [17] (rabbit polyclonal, 1:1000 dilution), anti-GFP (green fluorescent protein) (Roche #11 814 460 001, mouse monoclonal, 1:2000 dilution), anti-HIF1B (Cell Signaling Technology #5537, rabbit polyclonal, 1:1000 dilution), anti-GLUT1 (glucose transporter 1) (Thermo Scientific #RB-9052-P, rabbit polyclonal, 1:1000 dilution), anti-LDHA (lactate dehydrogenase A) (Cell Signaling Technology #2012, rabbit monoclonal, 1:1000 dilution), anti-CUL2 (cullin 2) (Invitrogen #51-1800, rabbit polyclonal, 1:2000 dilution) and anti-FLAG (Sigma #F7425, rabbit polyclonal, 1:1000 dilution).

Further methods can be found in the Supplementary Online Data at http://www.biochemj.org/bj/451/bj4510185add.htm.

## RESULTS

### USP52/PAN2 is a novel modifier of the hypoxic response

To identify new mediators of the HIF1A-mediated hypoxia pathway, we used U2OS osteosarcoma cells stably expressing a firefly luciferase reporter construct fused to three tandem copies of the iNOS (inducible nitric oxide synthase) HRE (hypoxia-response element) [19] (Figure 1A). U2OS-HRE cells responded to hypoxia (1% oxygen) by stabilization of HIF1A protein and concomitantly displayed an approximately 10-fold induction of luciferase activity (Figure 1A). The increase in luciferase activity was largely dependent on HIF1A, as siRNA-mediated depletion of HIF1A reduced luciferase activity to near-normoxic levels (Figure 1A). U2OS-HRE cells were screened with our custom-assembled ‘ubiquitome’ siRNA library targeting all known and assumed components of the ubiquitin and ubiquitin-like systems. On this basis, we identified a pool of siRNAs against USP52 that decrease hypoxia-dependent HRE-response activity. Deconvolution analysis revealed that two out of four individual siRNA duplexes targeting USP52 (si1 and si3) caused over a 40% reduction in luciferase activity, and this correlated closely with the reduction in USP52 protein by these siRNAs (Figure 1B). Interestingly, we were only able to deplete USP52 by approximately 50% as judged by immunoblot (Figure 1B) and real-time PCR (Supplementary Figure S1A at http://www.biochemj.org/bj/451/bj4510185add.htm) analysis. This is consistent with previous reports in mouse NIH 3T3 cells, where siRNA was reported to reduce USP52 by a maximum of ~65% compared with control siRNA [17]. *USP52* knockdown specifically impaired the hypoxia response, as it caused no such impairment to the NF-κB (nuclear factor κB) response after TNFα (tumour necrosis factor α) stimulation of the HeLa C57A cell line [20] (Supplementary Figure S1B). Next we generated U2OS-HRE cell lines stably expressing YFP-tagged USP52 resistant to either USP52 siRNA si1 or si3 (Figure 1C) and assessed the ability of siRNA-resistant USP52 to rescue the impaired hypoxic response upon endogenous *USP52* knockdown. Hypoxia-induced luciferase activity was rescued to approximately 80% and 95% in si1- and si3-resistant cells respectively, thus validating USP52 as a novel activator of the HIF1A-mediated hypoxic response (Figure 1D).

### USP52 potentiates the hypoxic response

**Figure 2:**
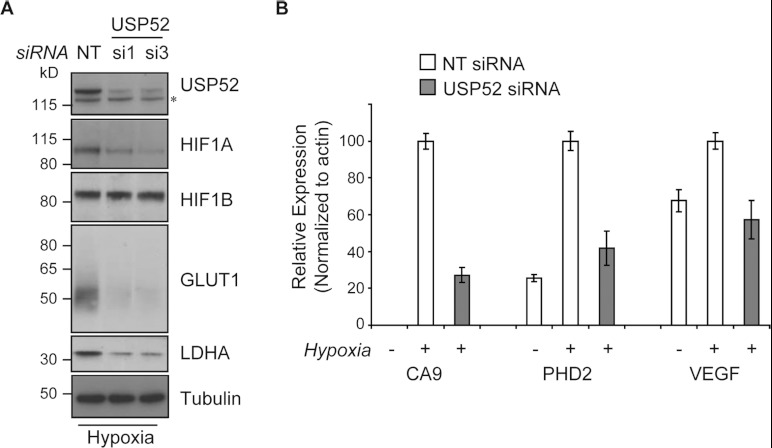
USP52 potentiates the hypoxic response (**A**) Depletion of USP52 using siRNA si1 or si3 in U2OS cells reduces the levels of HIF1A, but not HIF1B, protein as assessed by Western blot analysis. *HIF1A* transcriptional targets *GLUT1* and *LDHA* also showed decreased protein expression. Tubulin was used as a loading control. Molecular masses are indicated in kDa. (**B**) Real-time RT–PCR analysis shows that the expression of *HIF1A* targets *CA9*, *PHD2* and *VEGF* are all induced in U2OS cells in response to hypoxia. *USP52* knockdown reduces the ability of cells to increase expression of all *HIF1A* target genes. Levels were normalized to β-actin. Results are means±S.E.M.

To investigate further the role of USP52 in the HIF1A-mediated hypoxic response, U2OS cells were treated with individual USP52 siRNA duplexes 1 and 3 and exposed to hypoxia. Knockdown of *USP52* correlated with a specific decrease in HIF1A, but not HIF1B, protein levels (Figure 2A), suggesting that decreased HIF1A protein is responsible for the impaired hypoxia response. *GLUT1* and *LDHA* are both hypoxia-responsive genes which accumulate in U2OS cells in a HIF1A-dependent manner (Supplementary Figure S1C). Examination of GLUT1 and LDHA levels by immunoblot analysis of hypoxic U2OS cells revealed that USP52 depletion impairs the accumulation of GLUT1 and LDHA in response to hypoxia (Figure 2A), thus demonstrating that USP52 is required to potentiate the HIF1A-mediated hypoxic response. Similar results were obtained in mouse cells, indicating that USP52 has a conserved role in the hypoxia response (results not shown). Furthermore, real-time RT (reverse transcription)–PCR analysis from USP52-depleted U2OS cells demonstrated that the accumulation of the HIF1A target genes *CA9* (carbonic anhydrase IX), *PHD2* and *VEGF* (vascular endothelial growth factor) in response to hypoxia were all reduced (Figure 2B), confirming the role of USP52 in the HIF1A pathway. Interestingly, it has been reported previously that *USP52*/*KIAA0710* mRNA itself was induced 3.1-fold in response to hypoxia determined by microarray analysis of human renal cancer 786-O cells [21]. Whereas examination of the *USP52* promoter region revealed the presence of an HRE consensus motif (RCGTG) (Supplementary Figure S1D), no hypoxic increase in USP52 protein was detected in either 786-O or RCC4 cells (Supplementary Figure S1D), or in U2OS cells, HeLa cells or MEFs (mouse embryonic fibroblasts) (results not shown).

### USP52 regulates *HIF1A* mRNA

**Figure 3:**
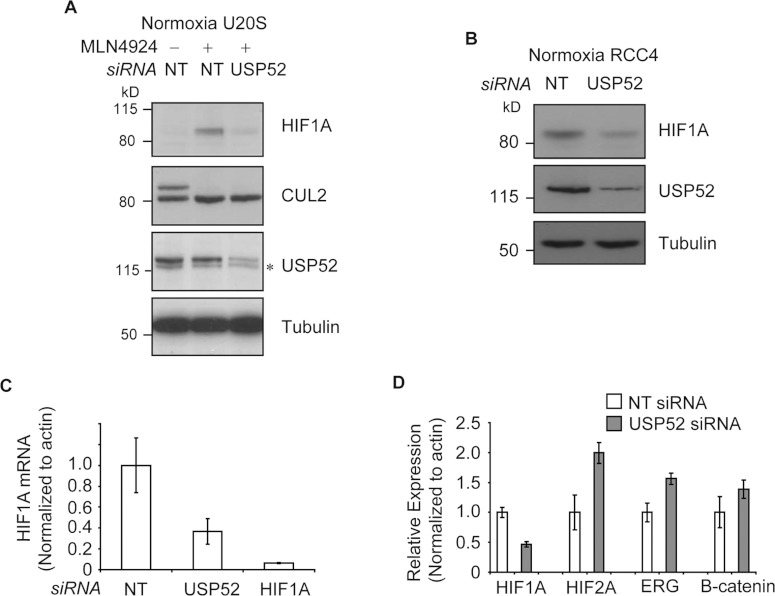
USP52 regulates the hypoxia pathway in a VHL-independent manner by controlling *HIF1A* mRNA levels (**A**) Normoxic U2OS cells were treated with MLN4924 to block CUL2 NEDD8ylation and inactivate the VHL complex. USP52 depletion was sufficient to decrease HIF1A protein levels, demonstrating independence of the VHL complex. Tubulin was used as a loading control. (**B**) USP52 depletion in VHL-deficient RCC4 renal cancer cells reduced HIF1A protein levels under normoxia. Tubulin was used as a loading control. (**C**) U2OS cells were treated with siRNA against *USP52* and *HIF1A*, and the levels of *HIF1A* mRNA were assessed by real-time RT–PCR analysis. *HIF1A* siRNA reduced *HIF1A* mRNA levels to below 10%, whereas USP52 depletion reduced *HIF1A* mRNA to approximately 40%. Levels were normalized to β-actin. (**D**) USP52 was depleted in HeLa cells which were subject to real-time RT–PCR analysis. *HIF1A* mRNA levels were reduced by approximately 50% in HeLa cells by *USP52* knockdown. *HIF2A* levels were increased 2-fold upon *USP52* knockdown, whereas *ERG* and *CTNNB1* mRNA levels were both increased 1.5-fold upon USP52 depletion. Levels were normalized to β-actin. Molecular masses are indicated in kDa in the blots, and results in histograms are means±S.E.M.

To gain insight into how USP52 may alter HIF1A protein levels, we performed protein network analysis of known USP52-interacting partners (Supplementary Figure S2A at http://www.biochemj.org/bj/451/bj4510185add.htm). Interestingly, USP52 was reported previously to immunoprecipitate TCE (transcription elongation factor) B1/elongin C and TCEB2/elongin B [22], which constitute part of the VHL E3 ligase complex required for the bulk of HIF1A degradation. The VHL complex is a cullin-type E3 ligase which requires CUL2 NEDD8ylation for ligase activity. To address whether USP52 negatively regulates HIF1A protein through interactions with the VHL complex, U2OS cells were treated with MLN4924 [23] to inhibit the NEDD8 (neural-precursor-cell-expressed developmentally down-regulated 8)-interacting enzyme and block VHL activity. Non-ubiquitylated HIF1A accumulated in normoxia upon MLN4924 treatment concomitant with the blockage of CUL2 NEDD8ylation (Figure 3A). However, depletion of USP52 caused a reduction in HIF1A protein even in the presence of MLN4924, suggesting that USP52 affects the HIF1A pathway independently of the VHL complex (Figure 3A). RCC4 cells lack functional VHL and therefore constitutively express HIF1A in normoxia [4]. In support of a VHL-independent role of USP52 in the HIF1A pathway, knockdown of *USP52* in RCC4 cells caused a subsequent decrease in *HIF1A* levels (Figure 3B). In addition to VHL, several other proteins have been reported to be involved in targeting HIF1A for proteasomal degradation, such as RACK1 (receptor for activated C-kinase 1) [24], HAF (hypoxia-associated factor) [25] and CHIP [C-terminus of the Hsc (heat-shock cognate) 70-interacting protein] [26]. In order to exclude *USP52* knockdown promoting *HIF1A* depletion through alternative degradation pathways, U2OS cells depleted of USP52 were treated with the proteasome inhibitor MG132. Proteasome inhibition caused the accumulation of ubiquitylated HIF1A (Supplementary Figure S2B) in NT siRNA-treated, but not USP52-depleted, cells, confirming that regulation of HIF1A by USP52 does not occur at the level of proteasomal degradation (Supplementary Figure S2B). To determine whether *USP52* knockdown alters *HIF1A* mRNA levels, real-time RT–PCR was performed on cDNA prepared from U2OS cells. USP52 depletion caused an approximately 60% reduction in the abundance of *HIF1A* mRNA (Figure 3C). As a control, *HIF1A* siRNA depleted *HIF1A* mRNA by over 90% (Figure 3C). To assess whether depletion of USP52 also affected *HIF2A* mRNA steady-state levels, HeLa cells were used as they express higher levels of HIF2A than U2OS cells. Whereas USP52 depletion caused a comparable reduction in *HIF1A* mRNA in HeLa cells, the levels of *HIF2A* actually increased approximately 2-fold (Figure 3D). This demonstrates that *USP52* knockdown specifically reduces *HIF1A* mRNA abundance, and supports the previous observation that decreased *HIF1A* mRNA is associated with up-regulation of HIF2A [19]. We also assessed the effect of USP52 depletion on mRNA levels of the unrelated genes *ERG* (Ets-related gene) and *CTNNB1* (β-catenin). Transcript levels of both *ERG* and *CTNNB1* were increased ~1.5-fold upon USP52 depletion (Figure 3D), consistent with the expected role of USP52 in mRNA degradation-dependent deadenylation. These results show that the role of USP52 in maintaining *HIF1A* mRNA levels displays a certain level of specificity.

### USP52 is required for *HIF1A* mRNA stability

**Figure 4:**
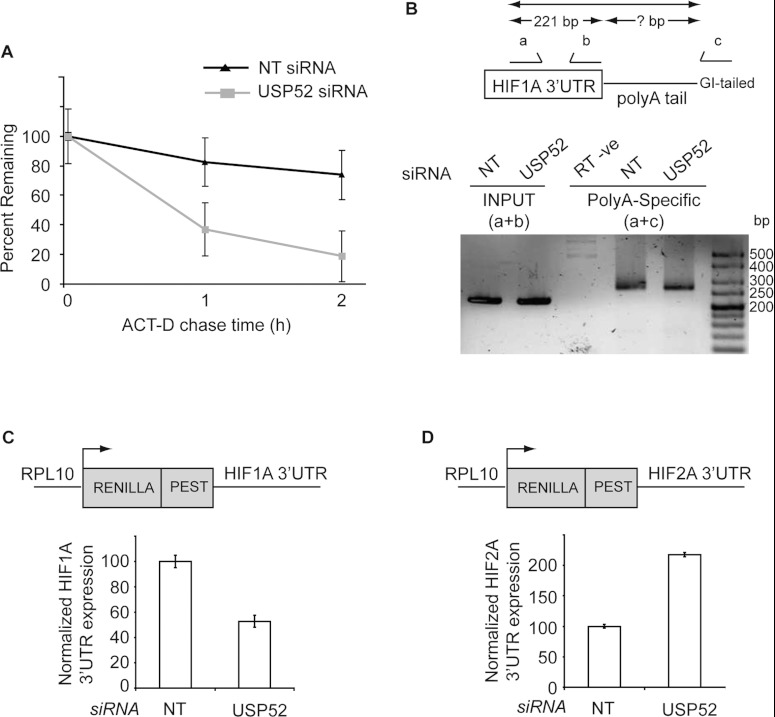
USP52 is required for stability of *HIF1A* mRNA (**A**) Actinomycin D (ACT-D) chase experiments were performed in U2OS cells treated with either NT or *USP52* siRNA. Real-time RT–PCR analysis revealed that depletion of USP52 decreased *HIF1A* mRNA half-life from 214 min to 35 min. Initial mRNA levels were normalized to 100% to account for lower *HIF1A* levels in USP52-depleted cells. Levels were normalized to β-actin. (**B**) U2OS cells were treated with NT or *USP52* siRNA, and cDNA was made from G/I-tailed mRNA. PCR was performed using primers within *HIF1A* 3′-UTR (a+b) to generate a 221 bp product, and in a separate reaction with forward primer (a) and universal reverse primer (c). Poly(A) tail length in both NT and *USP52* siRNA-treated cells was found to be predominantly ~60 residues long, calculated by subtracting the size of the (a+b) reaction product from the size of the (a+c) reaction product. Sizes are indicated in bp. (**C**) HEK-293 cells depleted of USP52 were transfected with the *HIF1A* 3′-UTR *Renilla* luciferase reporter shown. *USP52* knockdown caused a 50% decrease in the expression of *HIF1A* 3′-UTR construct. *Renilla* luciferase values were normalized to firefly luciferase to control for transfection efficiency. (**D**) HEK-293 cells depleted of USP52 were transfected with the *HIF2A* 3′-UTR *Renilla* luciferase reporter shown. *HIF2A* 3′-UTR expression was increased 2-fold upon USP52 depletion. *Renilla* luciferase values were normalized to firefly luciferase to control for transfection efficiency. Results are means±S.E.M.

USP52 depletion could modify the steady-state levels of *HIF1A* mRNA through modulating its transcription or stability. To distinguish between these possibilities, we performed actinomycin D chase experiments to measure the half-life of *HIF1A* mRNA after *USP52* knockdown. Actinomycin D inhibits RNA polymerase II to block transcription and therefore allows measurement of mRNA decay. Depletion of USP52 in U2OS cells dramatically enhanced the degradation of *HIF1A* mRNA (Figure 4A), where the half-life of HIF1A in NT-treated cells was 214 min (Supplementary Figure S3A at http://www.biochemj.org/bj/451/bj4510185add.htm) and reduced to 35 min upon USP52 depletion (Supplementary Figure S3B). In order to determine whether the effect of USP52 depletion on *HIF1A* mRNA stability was associated with a change in HIF1A poly(A) tail length, we analysed poly(A) tail length in U2OS cells treated with NT or *USP52* siRNA. Surprisingly, there was no apparent difference in the length of the *HIF1A* poly(A) tail after USP52 depletion, indicating that USP52 stabilizes *HIF1A* mRNA in a poly(A)-tail-length-independent manner (Figure 4B). The presence of multiple AREs in *HIF1A*'s 3′-UTR results in the regulation of HIF1A by AMD [7,8]. To determine whether USP52 regulates *HIF1A* stability through stabilizing its 3′-UTR, we used a *Renilla* luciferase reporter construct fused to the *HIF1A* 3′-UTR. Depletion of USP52 in HEK-293 cells inhibited *HIF1A* 3′-UTR activity by approximately 50% (Figure 4B), supporting a role for USP52 in *HIF1A* stabilization by preventing its AMD. Similar results were obtained upon *USP52* knockdown in U2OS cells (Supplementary Figure S3C). Interestingly, *USP52* knockdown resulted in an increase in the levels of *HIF2A Renilla* 3′-UTR in HEK-293 cells (Figure 4C), suggesting that the observed antagonism between HIF1A and HIF2A levels [19] (Figure 3D) may be regulated in a manner depending on the *HIF2A* 3′-UTR. To rule out any effects of USP52 on *HIF1A* translation, we performed polysome profiling on U2OS cells treated with either NT or *USP52* siRNA. There were no clear differences in the distribution of *HIF1A* mRNA between the non-translating and translating fractions (Supplementary Figure S4 at http://www.biochemj.org/bj/451/bj4510185add.htm), suggesting the effects of USP52 depletion are not mediated at the translation level. Collectively, these results demonstrate that USP52 regulates *HIF1A* mRNA stability in a 3′-UTR-dependent but poly(A)-tail-length-independent manner.

### USP52 is a component of cytoplasmic P-bodies

**Figure 5:**
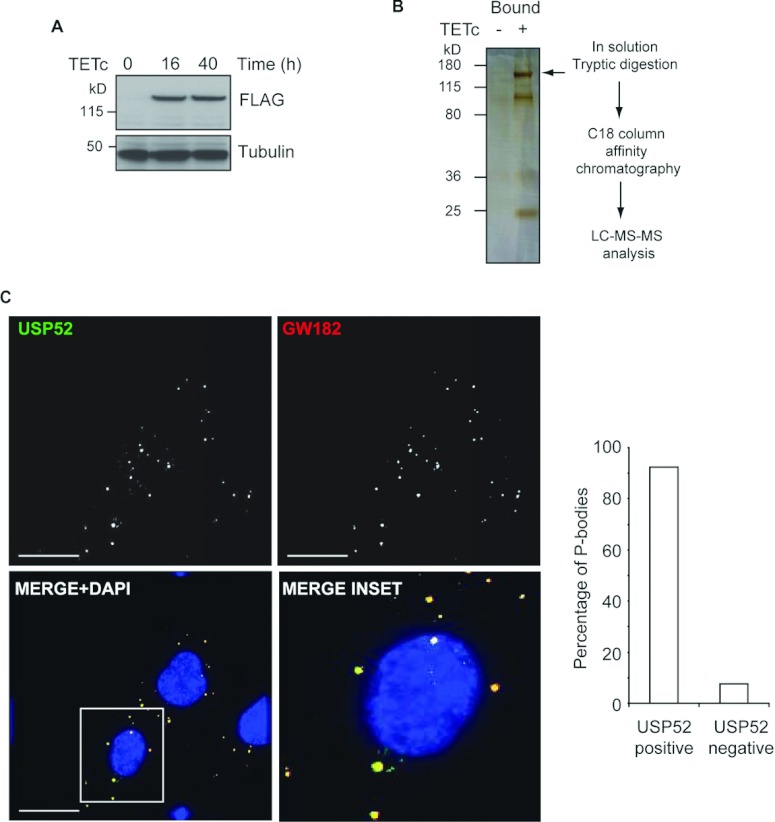
USP52 interacts with P-body components (**A**) HEK-293 cells expressing tetracycline (TETc)-inducible FLAG–USP52 were generated. *USP52* expression was induced by 16 or 40 h of TETc treatment as assessed by Western blot analysis. Tubulin was used as a loading control. (**B**) Inducible USP52 cells were either untreated or treated with TETc before FLAG–USP52 immunopreciptation. A fraction of eluted proteins (1/20th) were separated by denaturing gel electrophoresis and silver-stained. The position of FLAG–USP52 is indicated by an arrow. The remaining volume of eluted proteins was prepared for LC–MS/MS analysis as indicated in the flow chart. (**C**) Co-immunofluorescent staining of U2OS cells with USP52 and the P-body marker GW182 confirms that USP52 is a component of P-bodies. Quantification revealed that over 90% of P-bodies were USP52-positive, where 468 P-bodies were counted from 113 cells. Scale bars, 30 μm. Cells were counterstained with DAPI (4′,6-diamidino-2-phenylindole). Molecular masses are indicated in kDa in the blots.

To obtain insight into the observed USP52-mediated stabilization of *HIF1A* mRNA, we performed proteomic analysis of USP52 immunoprecipitates to identify new USP52-associated proteins. HEK-293 cells which stably express FLAG-tagged USP52 in a tetracycline-responsive manner were generated (Figure 5A). FLAG–USP52 expression was induced for 24 h before anti-FLAG-conjugated beads were used to immunoprecipitate FLAG–USP52 complexes. Immunoprecipitated material from induced and uninduced control cells was separated by denaturing gel electrophoresis and silver-stained to reveal the presence of specific USP52-associated proteins of various sizes (Figure 5B). The remaining material was subject to in-solution tryptic digestion and LC (liquid chromatography)–MS/MS (tandem MS) analysis to identify interacting proteins (Figure 5B). The full dataset is listed in Supplementary Table S4 at http://www.biochemj.org/bj/451/bj4510185add.htm. We were interested by the identification of the P-body components TRIM21 (tripartite motif-containing 21), PCBP1 and PAN3 in USP52 immunoprecipitates (Supplementary Table S4), given the role of P-bodies in mRNA storage and/or degradation [27]. To confirm that USP52 is a component of P-bodies [18], immunostaining of U2OS cells with antibodies against USP52 and the essential P-body component GW182 demonstrated that USP52 co-localizes to P-bodies (Figure 5D). Quantification revealed that 92.5% of GW182 P-bodies were also USP52-positive (Figure 5D). Treatment of cells with CHX or puromycin is known to abolish or increase respectively the number of P-bodies [27]. We confirmed that USP52 P-body foci were likewise abolished by CHX treatment, while the number increased upon puromycin treatment (Supplementary Figure S5A at http://www.biochemj.org/bj/451/bj4510185add.htm). This confirmed that USP52 is a genuine P-body component and raised the possibility that USP52 may serve a role in regulating *HIF1A* mRNA through its association with P-bodies.

### Disrupting USP52-containing P-bodies by GW182 depletion reduces *HIF1A* mRNA

**Figure 6:**
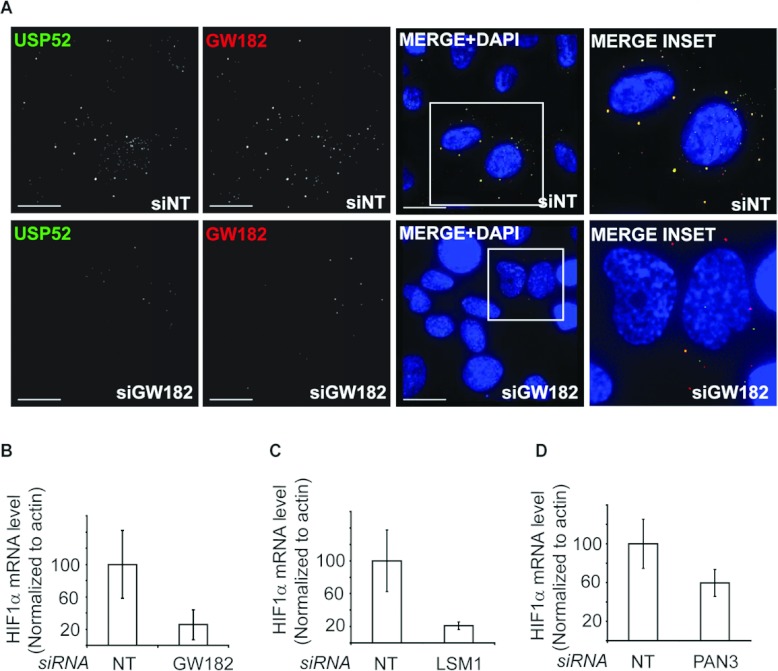
GW182 maintains P-body integrity and *HIF1A* mRNA levels (**A**) U2OS cells treated with either NT or *GW182* siRNA were co-immunostained with USP52 and GW182. GW182 depletion by siRNA reduced the total number of P-bodies and dispersed USP52 from foci. Scale bars, 30 μm. Cells were counterstained with DAPI (4′,6-diamidino-2-phenylindole). (**B**) GW182 depletion caused a reduction in *HIF1A* mRNA levels to approximately 20%. Levels were normalized to β-actin. (**C**) LSM1 depletion caused a reduction in *HIF1A* mRNA levels. (**D**) PAN3 depletion caused a reduction in *HIF1A* mRNA levels. Levels were normalized to β-actin. Results in histograms are means±S.E.M.

The co-localization of USP52 with P-bodies led us to hypothesize that P-bodies might be important for *HIF1A* mRNA stability. GW182 is essential for P-body integrity, as its depletion abolishes the presence of visible P-bodies [28]. To determine whether P-body disruption correlated with changes in *HIF1A* mRNA levels, we depleted GW182 from U2OS cells. *GW182* siRNA caused a decrease in the proportion of cells containing P-bodies, whereas any remaining P-bodies were visibly smaller (Figure 6A). USP52 foci were dispersed upon GW182 depletion, confirming that the presence of USP52-positive P-bodies is dependent on GW182 (Figure 6A) and quantification of P-bodies revealed that the percentage of cells containing at least one P-body decreased from 80% in NT siRNA-treated cells to approximately 20% in *GW182* siRNA-treated cells (Supplementary Figure 5B). Importantly, we observed a reduction in *HIF1A* mRNA levels of almost 80% upon GW182 depletion (Figure 6B), suggesting that P-body integrity plays an important role in maintaining *HIF1A* mRNA levels. Whereas GW182 depletion dispersed USP52-positive P-bodies, it had no effect on steady-state levels of USP52 protein, suggesting that *HIF1A* mRNA levels were not affected by simply reducing the amount of USP52 protein (Supplementary Figure S5C). As GW182 has also been proposed to be involved in regulating the miRNA (microRNA) pathway, it was important to confirm that dispersing P-bodies by depleting another P-body component independent of the miRNA pathway had the same effect on *HIF1A* mRNA. We therefore depleted LSM1, which dispersed P-bodies (Supplementary Figure S5C) while also resulting in a reduction of *HIF1A* mRNA to approximately 20% (Figure 6C). PAN3 has been shown to recruit USP52 to P-bodies, and its depletion was also shown to reduce P-body number as well as destabilize AMD substrates [18]. We therefore depleted PAN3 in U2OS cells and observed that *HIF1A* mRNA levels were decreased to 60% compared with NT siRNA (Figure 6D). Altogether, these results suggest that P-bodies are important regulators of steady-state *HIF1A* mRNA levels, and that USP52 is a key component of P-bodies required for *HIF1A* mRNA stability.

### *HIF1A* mRNA is present in P-bodies

**Figure 7:**
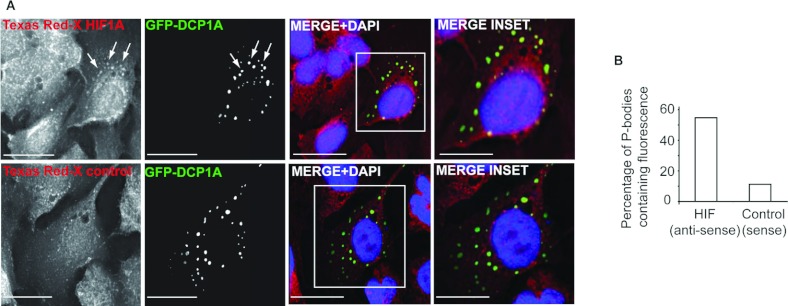
*HIF1A* mRNA localizes to P-bodies (**A**) U2OS cells were transfected with GFP–DCP1A, and *HIF1A* mRNA localization was assessed by FISH analysis with Texas-Red-X-labelled antisense probes. HIF1A was found to co-localize to GFP–DCP1A-positive P-bodies, whereas only background co-localization was observed with sense control probes. Scale bars, 30 μm. Nuclei were counterstained with DAPI (4′,6-diamidino-2-phenylindole). (**B**) The number of GFP–DCP1A-positive P-bodies containing HIF1A fluorescence (*n*=284) compared with sense control (*n*=489) were determined.

The finding that USP52 is a P-body component required for *HIF1A* mRNA stability coupled to our finding that reducing the number of cellular P-bodies depletes *HIF1A* mRNA raised the possibility that *HIF1A* mRNA might be present within P-bodies. To test this directly, we performed FISH (fluorescent *in situ* hybridization) with antisense Texas-Red-X-labelled probes specific to *HIF1A* mRNA on U2OS cells transfected with the P-body marker GFP–DCP1A (decapping enzyme 1A). A GFP-tagged P-body marker was used to circumvent problems associated with the destruction of endogenous epitopes upon harsh denaturing conditions required for FISH. *HIF1A* antisense probes formed discrete cytoplasmic foci which co-localized with GFP–DCP1A-positive P-bodies (Figure 7A), suggesting that *HIF1A* mRNA is a P-body constituent. Non-specific fluorescence was deduced by using *HIF1A* sense control probes, which did not show an obvious co-localization with GFP–DCP1A (Figure 7A). The proportion of GFP–DCP1A P-bodies co-localizing with *HIF1A* antisense probes was calculated to be 52%, whereas antisense probes showed a 10% co-localization rate (Figure 7B), probably due to background fluorescence showing coincidental overlap with GFP–DCP1A. Therefore a proportion of *HIF1A* mRNA is found within P-bodies, suggesting that these may act as storage sites for *HIF1A* mRNA.

## DISCUSSION

HIF1A is the master regulator of the cellular response to hypoxia which is responsible for executing a transcriptional programme endowing cells with the capacity to deal with hypoxic stress [3]. Whereas the oxygen-dependent regulation of HIF1A protein has been well described, it is becoming increasingly apparent that regulation of *HIF1A* mRNA also plays an important role in regulating HIF1A-dependent processes. In the present study, we have identified the P-body component USP52/PAN2 as an important stability factor for *HIF1A* mRNA, and have demonstrated that it prevents degradation of *HIF1A* mRNA in a 3′-UTR-dependent, but poly(A)-tail-length-independent, manner.

P-bodies are dynamic structures which are important in both mRNA decay and miRNA-mediated translational silencing, acting as a store for mRNAs before their release back into active polysomes [27]. It is clear that miRNA pathways are important regulators of the hypoxic response and that several miRNAs are up-regulated in response to hypoxia [29]. In addition, a *Drosophila* genome-wide siRNA screen identified several components of the miRNA machinery that were required for a complete hypoxic response, including the P-body components Ago1 and GW182 [30]. The fact that P-bodies are reported to be a site of ARE-containing mRNA storage and AMD [31] suggested that *HIF1A* mRNA may be stored and/or degraded in P-bodies. Our finding that depletion of the P-body component USP52 destabilizes *HIF1A* mRNA via its AU-rich 3′-UTR suggests that USP52 plays a key role in preventing AMD of *HIF1A* mRNA in P-bodies. This may occur through preventing the association of *HIF1A* 3′-UTR with AMD-promoting proteins such as TTP, which is present in P-bodies and known to promote the degradation of ARE-containing mRNAs including *HIF1A* [7,8,31]. Although we cannot rule out the possibility of indirect effects of USP52 on *HIF1A* mRNA stability, our finding that *HIF1A* mRNA and USP52 protein both reside in P-bodies coupled with the fact that abolishing visible P-bodies also destabilizes *HIF1A* mRNA supports a more direct role for USP52 in regulating *HIF1A* mRNA.

Deadenylation of mRNA occurs through the concerted actions of the PAN2–PAN3 and CCR4–CAF1 complexes [17], and is a necessary first step in all major mRNA decay pathways including AMD and miRMD (miRNA-mediated decay) [32]. Although USP52/PAN2 is dispensable in yeast [12], consistent with the fact that CCR4 provides the major cellular deadenylation activity [16], our finding that USP52 is required for *HIF1A* mRNA stability was surprising. However, this apparent paradox may be explained by USP52 playing additional roles to deadenylation when localized in P-bodies, which is supported by the finding that PABPC1 (which is required for USP52 nuclease activity) is absent from P-bodies [18,33]. Indeed, we did not observe any effect of USP52 depletion on the length of the *HIF1A* poly(A) tail (Figure 4B), and it is consistent with our finding that, whereas USP52 depletion destabilized *HIF1A* mRNA, overexpression of a nuclease-dead USP52 was as efficient as wild-type USP52 in enhancing the HIF1A-mediated hypoxic response (results not shown). Interestingly, it has also been reported that PAN3 depletion causes a reduction in P-body number concomitant with a destabilization of ARE-containing transcripts [18]. Furthermore, these authors showed that PAN3 was able to recruit USP52/PAN2 to P-bodies [18]. It is therefore a possibility that PAN3 depletion prevents USP52 localization to P-bodies and thus contributes to the destabilization of AMD substrates, as we have observed in the case of HIF1A. In this scenario, USP52 may regulate the stability of other ARE-containing transcripts in addition to HIF1A.

USP52 has also been implicated in miRNA pathways, as it was shown to be recruited by GW182 through interactions with PAN3 in humans, *Drosophila* and *Caenhorhabditis elegans* [34–37]. It is therefore possible that GW182 recruits USP52 to P-bodies through interactions with PAN3 [18]. We observed that P-body dispersal correlates with decreased levels of *HIF1A* mRNA, suggesting that the degradation of *HIF1A* mRNA is prevented by USP52 within P-bodies. This is consistent with previous observations that ARE-containing transcripts are recruited to and stored in P-bodies [31], and we confirm further that *HIF1A* mRNA is present within P-bodies [38,39]. In addition, it was shown that genetic disruption of GW182 in *Drosophila* dramatically reduced the mRNA levels of the *HIF1A* homologue *Sima* in response to hypoxia [30], suggesting that there is a highly conserved role for P-bodies in maintaining *HIF1A* mRNA levels.

USP52 exhibits homology with the ubiquitin-specific protease family, but is not an active DUB (deubiquitinating enzyme) owing to mutations in two of the three residues of the catalytic triad [11]. We therefore were initially interested in the possible role of USP52 as a pseudo-DUB. Pseudo-DUBs may be predicted to act in a dominant-negative manner preventing other DUBs from gaining access to their substrate, or alternatively they may bind ubiquitylated proteins and play a scaffolding role in protein–protein interactions. Interestingly, we found several peptides corresponding to ubiquitin in our USP52 immunoprecipitates, and transiently overexpressing USP52 harbouring a Cys-box deletion mutation in the UCH (ubiquitin C-terminal hydrolase) domain resulted in a decrease in HIF1A protein (results not shown), suggesting that this domain may be important in protecting *HIF1A* mRNA in P-bodies. Interestingly, it has recently been reported that the RNA-binding E3 ubiquitin ligase MEX-3C is required for the RING-dependent 3′-UTR-mediated degradation of *HLA-A2* mRNA, suggesting that there is a wider-role for ubiquitin-handling proteins in mRNA stability pathways [40]. Intriguingly, we identified the E3 ubiquitin ligase and P-body component TRIM21 as a USP52-associated protein, opening the possibility that USP52 may be involved in regulating the function of TRIM21 or its substrates. Future studies exploring the potential role of USP52 as a pseudo-DUB in its role in P-body function will be useful in expanding the understanding of ubiquitin-mediated regulation of RNA metabolism.

## Online data

Supplementary data
