# Supplementary material for: The P-body component USP52/PAN2 is a novel regulator of HIF1A mRNA stability
Source: Biochem J. 2013 Mar 28;451(Pt 2):185–94. doi: 10.1042/BJ20130026 (PMC3632086; doi:10.1042/BJ20130026)
Supplement: Supplementary data [file bj4510185add.pdf]

BJ Cell

Biochem. J. (2013) 451, 185–194 (Printed in Great Britain) doi:10.1042/BJ20130026

online data

SUPPLEMENTARY ONLINE DATA

The P-body component USP52/PAN2 is a novel regulator of *HIF1A* mRNA stability

John S. BETT*, Adel F. M. IBRAHIM*, Amit K. GARG*, Van KELLY*, Patrick PEDRIOLI*, Sonia ROCHA† and Ronald T. HAY*†[1]

*Scottish Institute for Cell Signalling, Sir James Black Centre, College of Life Sciences, University of Dundee, Dow Street, Dundee DD1 5EH, Scotland, U.K., and
†Wellcome Trust Centre for Gene Regulation and Expression, College of Life Sciences, University of Dundee, Dundee DD1 5EH, Scotland, U.K.

EXPERIMENTAL

Plasmids

The *Renilla* luciferase–*HIF1A*-3′-UTR and *Renilla* luciferase–*HIF2A*-3′-UTR plasmids were purchased from Switchgear Genomics. Plasmid pcDNA5-FRT/TO-FLAG-USP52 was made by amplifying USP52 isoform 3 from a human cDNA library (with primers forward 5′-GCGGCCGCCATGAACTTTGAGGGT-3′ and reverse 5′-GCGGCCGCTCAGAGCGCCAGCACT-3′) and using standard cloning techniques. The pEFIRES-B-eYFP vector was created by inserting the eYFP (enhanced YFP) sequence from pEYFP-C1 (Clontech) into the multiple cloning site of pEFIRES-P [37] and replacing the puromycin selection marker with a blasticidin resistance marker. The pEFIRES-B-eYFP-USP52 plasmid was generated by PCR amplification of USP52 (isoform 1) from an IMAGE clone (with primers forward 5′- GT-AGATCTATGAACTTTGAGGGTCTGGACC-3′ and reverse 5′-GTGCGGCCGCTCAGAGCGCCAGCACTGAGGAG-3′) and using standard cloning techniques. YFP–USP52 siRNA-resistant forms were generated by standard mutagenesis protocols to make silent mutations (si1-resistant mutant g1545a; t1548c; t1551c and si3-resistant mutant g2634a; c2640a; t2643c). pcDNA5-FRT/TO-GFP-DCP1A was generated by amplifying DCP1A from IMAGE clone (3029175) with primers (forward 5′-GGGGCGGCCGCGATGGAGGCGCTGAGTCGAGCTGGG-3′ and reverse 5′- GGGGCGGCCGCTCATAGGTTGTGGTTGT-CTTTGTT-3′) and using standard cloning techniques. All DNA transfections were carried out with Lipofectamine™ 2000 according to the manufacturer's instructions.

RNA, real-time RT–PCR and poly(A) tail length analysis

Total RNA was extracted using the RNeasy Mini Kit (Qiagen) and cDNA synthesis was performed with First Strand cDNA synthesis kit (Fermentas) according to the manufacturer's instructions. USP52 real-time assays were performed using PerfeCta™ SYBR Green fast mix (Quanta Biosciences) at 95 °C for 10 min, and 45 cycles of 15 s at 95 °C and 1 min at 58 °C. All other real-time RT–PCR was performed using probe-based Solaris assays (Thermo Scientific) using the manufacturer's recommended reactions and cycling conditions. All of the assays used gave standard curves giving between 90 and 105 % efficiency and R^2 values of 0.99, and were performed on a Bio-Rad CFX96 real-time system and analysed using CFX Manager software version 1.5 (Bio-Rad Laboratories). All assays were performed with biological triplicates and technical duplicates. Primer and probe sequences are given in Table S2. Actinomycin D chase experiments were performed by treating cells with 1 μg/ml actinomycin D (Sigma) for the indicated time after 48 h of siRNA treatment. Values were normalized to 100 % in untreated cells and the percentage remaining was calculated after actinomycin D treatment. Poly(A) tail length was calculated using the Poly(A) Tail Length Assay Kit (Affymetrix). Total RNA was harvested from U2OS cells treated with either NT or *USP52* siRNA, poly(A) tails were G/I-tailed and converted into cDNA according to the manufacturer's instructions. Forward and reverse primers were designed in the *HIF1A* 3′-UTR (5′-TTATGCACTTTGTCGCTATTAAC-3′ and 5′-GCCTGGTCCACAGAAGATG-3′ respectively) to generate a 221 bp product. Forward primer and the supplied universal reverse primer were then used to generate a product whose size was 221 bp plus the poly(A) tail length, thus allowing calculation of poly(A) tail length. Products were analysed on a 2.5 % agarose gel.

IP (immunoprecipitation) and MS

For IP experiments, induced and uninduced T-REx FLAG–USP52 cells were lysed in IP buffer [50 mM Tris/HCl (pH 7.4), 150 mM NaCl, 1 mM EDTA, 1 % (w/v) Triton X-100 and protease inhibitor cocktail (Sigma)] and anti-FLAG M2 affinity gel (Sigma, #A2220) was used to precipitate FLAG-tagged USP52 according to the manufacturer's instructions. Beads were washed five times with IP buffer, then a further five times with IP buffer lacking detergent. Proteins were sequentially eluted with 0.2 M glycine (pH 2.5) then 8 M urea (pH 8) and buffered in 10 mM ammonium bicarbonate. Eluates were reduced with 5 mM TCEP [tris-(2-carboxyethyl)phosphine] (45 min at 37 °C), alkylated with 15 mM iodacetamide [45 min at room temperature (20 °C)] and digested with trypsin overnight at 37 °C. Peptides were acidified to pH <3.0 with TFA (trifluoroacetic acid) and purified on C_{18} microspin columns (Nest Group) before MS analysis. Samples were analysed by LC–MS/MS on an LTQ Orbitrap Velos instrument (Thermo Fisher Scientific). Data were analysed using Mascot (http://www.matrixscience.com). Peptides found in control only cells were assumed to be non-specific contaminants and disregarded.

Immunofluorescence, FISH and deconvolution microscopy

Immunofluorescent labelling was carried out on U2OS cells grown on Lab-Tek® chamber slides (Nunc). Cells were fixed with 5 % (w/v) formaldehyde for 10 min and permeabilized with methanol for 5 min before blocking in DMEM (Dulbecco's modified Eagle's medium) containing 10 % (w/v) FBS for 1 h. Primary and secondary antibodies were diluted in block, and incubations were carried out for 1 h each. Secondary antibodies were Cy5 (indodicarbocyanine)-conjugated IgG from Jackson ImmunoResearch Laboratories, and Alexa Fluor® 488-conjugated IgG from Invitrogen. Cells were counterstained with DAPI (4′,6-diamidino-2-phenylindole) (Sigma) for 10 min, mounted in Fluorescent Mounting Medium (Dako) and

[1] To whom correspondence should be addressed (email R.T.Hay@dundee.ac.uk).

© 2013 The Author(s)
The author(s) has paid for this article to be freely available under the terms of the Creative Commons Attribution Licence (CC-BY) (http://creativecommons.org/licenses/by/3.0/) which permits unrestricted use, distribution and reproduction in any medium, provided the original work is properly cited.

Biochemical Journal www.biochemj.org

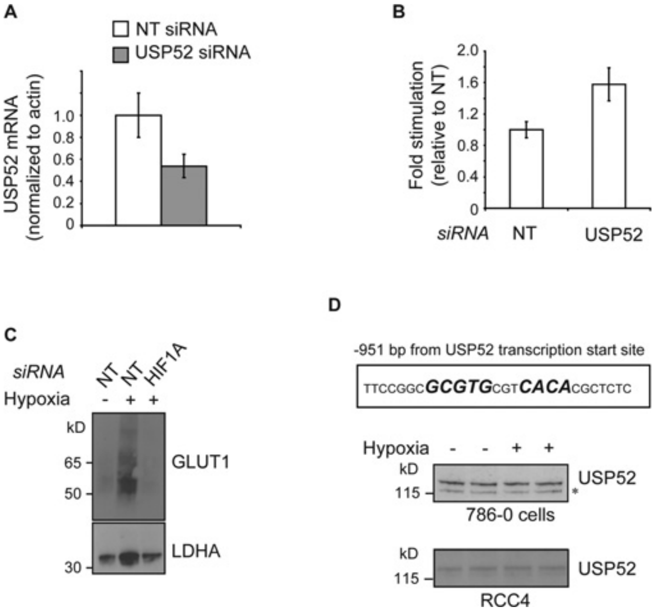

Figure S1 USP52 and hypoxia

(**A**) *USP52* siRNA reduces the level of *USP52* mRNA to approximately 50 % that by NT siRNA in U2OS cells. Levels were normalized to β-actin. (**B**) HeLa cells expressing an NF-κB (nuclear factor κB)-dependent luciferase promoter were stimulated with TNFα (tumour necrosis factor α) in duplicate. USP52 depletion did not cause impairment in the NF-κB-dependent response to TNFα stimulation. (**C**) Immunoblot analysis demonstrates that *GLUT1* and *LDHA* are both up-regulated in U2OS cells in a HIF1A-dependent manner upon exposure to 24 h of hypoxia. (**D**) USP52 contains an HRE in its regulatory region. USP52 is not induced by hypoxia in 786-O or RCC4 renal cancer cells. Molecular masses are indicated in kDa in the blots, and results in histograms are means$\pm$S.E.M.

coverslipped. Primary antibodies used were anti-USP52 [28] (rabbit polyclonal, 1:1000 dilution) and anti-GW182 (human autoantigen, 1:6000 dilution). Stained cells were viewed on a Delta Vision DV3 deconvolution microscope with an oil-immersion ×40 or ×63 objective lens and images were processed using Softworx (Applied Precision). Images presented are maximal intensity projections from deconvolved three-dimensional images. The proportion of GW182 P-bodies also containing USP52 was calculated by counting 468 P-bodies from 113 cells in deconvolved image projections of each field. The percentage of P-bodies per cell in NT or *GW182* siRNA-treated cells was calculated by counting the proportion of cells containing at least one P-body. At least 185 cells were counted for each condition. For FISH experiments, U2OS cells were transfected with GFP–DCP1A for 24 h, then fixed in 5 % formaldehyde for 10 min. Cells were permeabilized in 70 % ethanol overnight then rehydrated in 50 % formamide and 2× SSC (0.3 M NaCl/0.03 M sodium citrate) for 10 min. Next antisense or control sense Texas-Red-X (Invitrogen)-labelled probes (sequences given in Table S3) were diluted at 10 ng/μl in hybridization buffer (50 % formamide, 2× SSC, 2 mM vanadyl ribonucleoside complexes, 100 μg/ml total yeast RNA, 0.02 % BSA and 0.1 mg/ml dextran sulfate) and cells were incubated with diluted probes in a humid 37 °C chamber overnight. Cells were washed in 50 % formamide and 2× SSC twice for 30 min each before counterstaining with DAPI and coverslipping. Microscopy was as described above and at least 280 P-bodies per condition (antisense and sense) of projected deconvolved images carried out were counted.

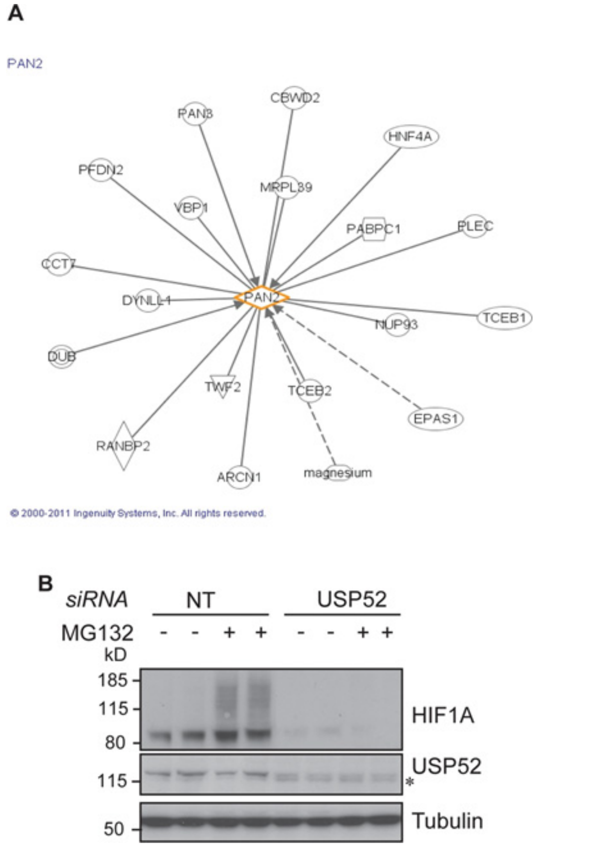

Figure S2 USP52 regulates HIF1A independently of proteasomal degradation

(**A**) Ingenuity Pathway Analysis was employed to reveal USP52-interacting proteins and regulators, including TCEB2/elongin B and TCEB1/elongin C. (**B**) U2OS cells treated with *USP52* siRNA were exposed to 24 h of hypoxia and treated with the proteasome inhibitor MG132. HIF1A protein levels were not rescued upon proteasome inhibition, demonstrating that USP52 does not alter HIF1A protein catabolism. Tubulin was used as a loading control. Molecular masses are indicated in kDa.

Table S1 Sequence of siRNAs

siRNA	Sequence (5′→3′)
USP52 (si1)	GACCUUGUUUGCUGGAUUA
USP52 (si2)	UCAAGGGUCUUUAUGAGAA
USP52 (si3)	GCAAGGAGGGCGUUACUCA
USP52 (si4)	AAGAACAACCUCAAGUAUA
GW182 (si1)	GCCUAAAUAUUGGUGAUUA
GW182 (si2)	GAACAAACUGCCUAGCAAU
GW182 (si3)	CAGUUUAUGUCCAGUCAAA
GW182 (si4)	CCGGCJUCAGUGCAGAAUA
LSM1 (si1)	CGAGAUGGAAGGACACUUA
LSM1 (si2)	GCGUAUUCAUGUGGGCAAA
LSM1 (si3)	GCAAGUAUCCAUUGAAGAA
LSM1 (si4)	GAGCAGAUACUCUUGAUGA
PAN3 (si1)	AAAACAAGGUUGCGAGUAA
PAN3 (si2)	CGACUUACUUCUAUACAGA
PAN3 (si3)	GGUUUGGCAUGUCGAGUUA
PAN3 (si4)	GGGCAUAUAUUGUCCAACU

© 2013 The Author(s)
The author(s) has paid for this article to be freely available under the terms of the Creative Commons Attribution Licence (CC-BY) (http://creativecommons.org/licenses/by/3.0/) which permits unrestricted use, distribution and reproduction in any medium, provided the original work is properly cited.

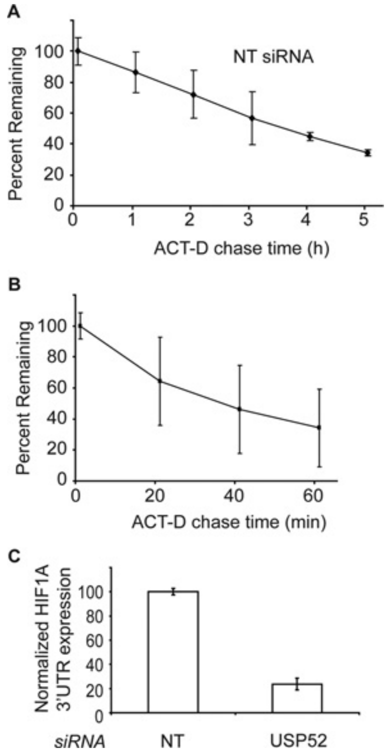

Figure S3 USP52 depletion destabilizes *HIF1A* mRNA

(**A**) Actinomycin D chase experiment in NT siRNA-treated U2OS cells reveals the HIF1A half-life to be 214 min in a 5 h time course. (**B**) Actinomycin D chase experiment in USP52-depleted U2OS cells reveals the H1F1A half-life to be 35 min over a 60 min time course. (**C**) USP52 depletion in U2OS cells caused an 80 % reduction in the expression of *Renilla* luciferase–*HIF1A*-3′-UTR. Levels were normalized to firefly luciferase. Results are means±S.E.M.

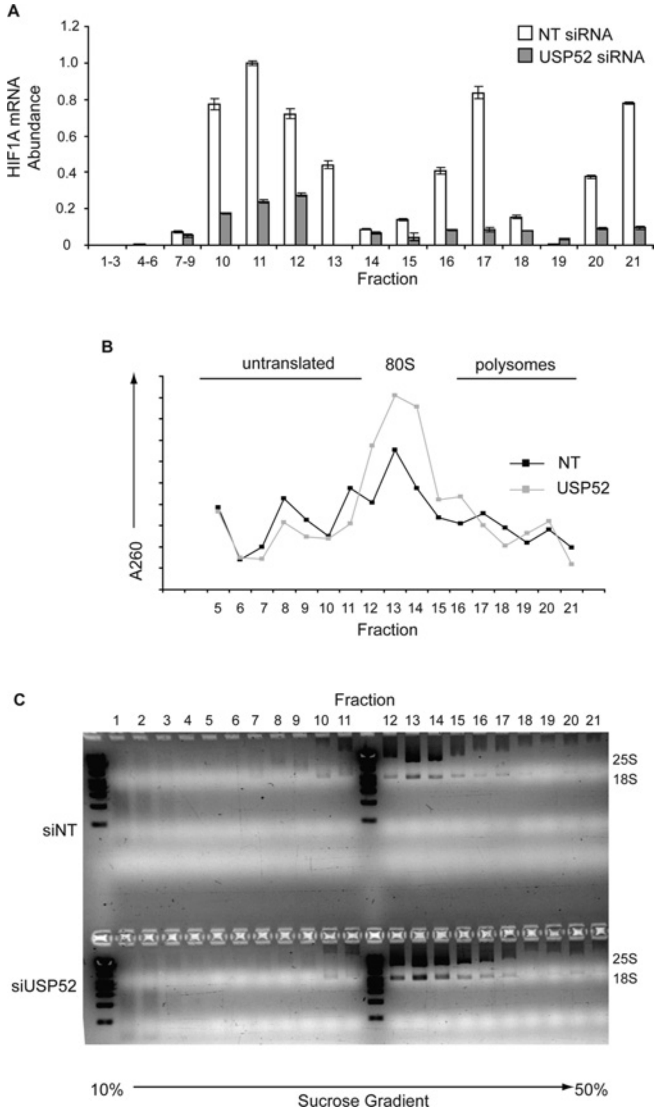

Figure S4 Polysome profile of USP52-depleted cells

(**A**) Real-time RT–PCR analysis of *HIF1A* mRNA in polysome profile fractions of NT or *USP52* siRNA-treated cells. Samples were prepared using a standard polysome profile protocol where cytosolic lysates were centrifuged at 36 000 rev./min for 3 h using an SW41 Ti swing-out rotor through a 10–50 % sucrose gradient (prepared at 10 % intervals), and 0.5 ml fractions were collected for RNA extraction and real-time RT–PCR analysis. (**B**) A_{260} values were taken for each fraction and plotted. Fraction 13 corresponds to 80S ribosomal RNA, heavier fractions (14–21) correspond to polysomes and lighter fractions (12 and below) correspond to the untranslated pool. (**C**) Fractions were run on an agarose gel and stained with ethidium bromide to enable RNA to be visualized.

© 2013 The Author(s)
The author(s) has paid for this article to be freely available under the terms of the Creative Commons Attribution Licence (CC-BY) (http://creativecommons.org/licenses/by/3.0/) which permits unrestricted use, distribution and reproduction in any medium, provided the original work is properly cited.

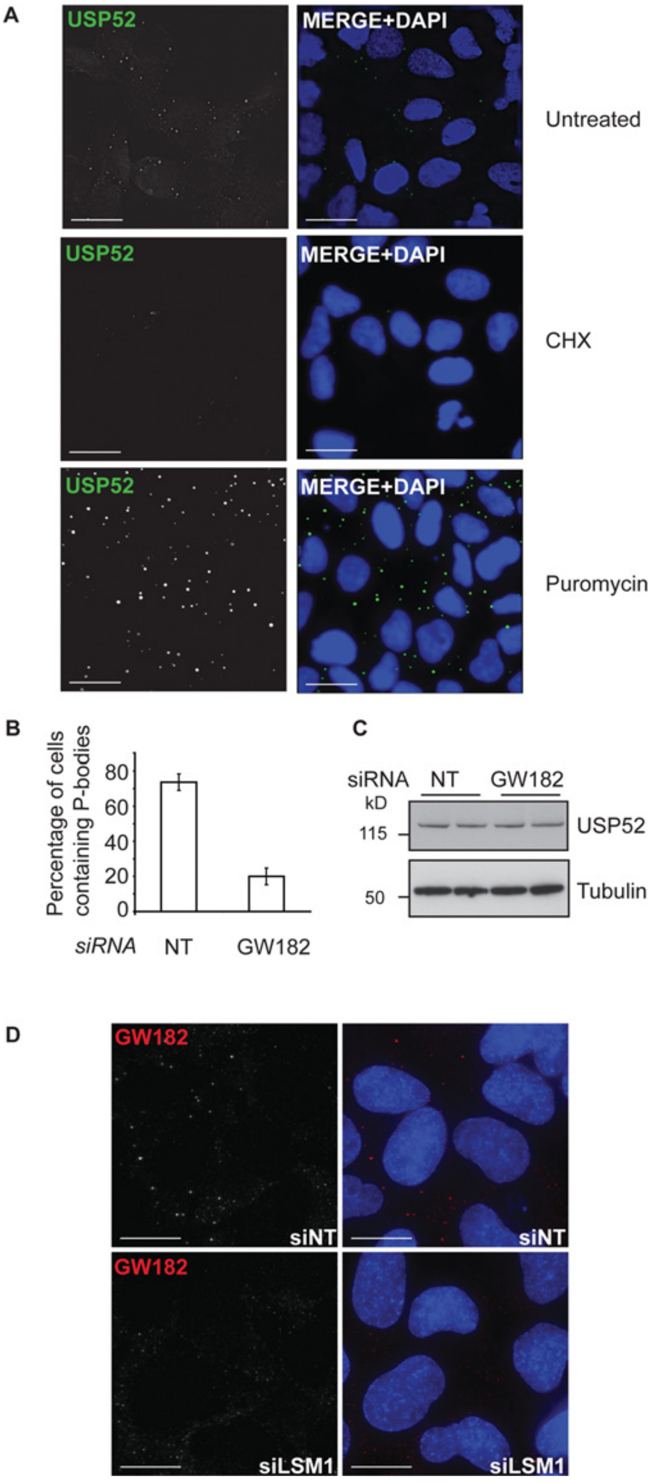

Figure S5 USP52 is a P-body component

(**A**) U2OS cells were treated with puromycin or CHX and immunostained with anti-USP52 antibody. USP52-positive foci were increased upon puromycin treatment and decreased upon CHX treatment confirming USP52 as a P-body component. Scale bars, 30 μm. Cells were counterstained with DAPI (4′,6-diamidino-2-phenylindole). (**B**) Quantification of the experiment shown in Figure 6(A) of the main text. *GW182* siRNA reduced the percentage of cells containing GW182- and USP52-positive P-bodies from approximately 80 % in NT-treated cells ($n = 226$) to 20 % in *GW182* siRNA-treated cells ($n = 185$). Results are means$\pm$S.E.M. (**C**) U2OS cells were treated with *GW182* siRNA and USP52 protein levels were found to be unchanged. Tubulin was used as a loading control. Molecular masses are indicated in kDa. (**D**) U2OS cells were depleted of LSM1 and P-bodies were immunostained with anti-GW182 antibody. P-bodies were dispersed upon LSM1 treatment. Cells were counterstained with DAPI. Scale bar, 15 μm.

Table S2 Real-time PCR primer and probe sequences

ACTB, β-actin.

Gene	Primer/probe	Sequence ($5' \rightarrow 3'$)
USP52 (SYBR)	Forward primer	ATGAGAAGGGCAGAAAGATGG
USP52 (SYBR)	Reverse primer	GGGCTATAGAACAGTAAAGGGAG
ACTB (SYBR)	Forward primer	CCCAGCACAATGAAGATCAAG
ACTB (SYBR)	Reverse primer	GACTCGTCATACTCCTGCTTG
HIF1A (Solaris)	Forward primer	TTACCATGCCCCAGATTCAG
HIF1A (Solaris)	Reverse primer	GGACTATTAGGCTCAGGT
HIF1A (Solaris)	Probe	GCACTAGACAAAGTTCACC
ACTB (Solaris)	Forward primer	TGGAGAAAATCTGGCACCAC
ACTB (Solaris)	Reverse primer	GGTCTCAAACATGATCTGG
ACTB (Solaris)	Probe	ACCGCGAGAAGATGACC
CA9 (Solaris)	Forward primer	TGAGTGCTAAGCAGCTCCA
CA9 (Solaris)	Reverse primer	CCATTCAAAGGCTGCGT
CA9 (Solaris)	Probe	TGAACTTCCGAGCGACG
HIF2A (Solaris)	Forward primer	ATGGGACTTACACAGGTGGA
HIF2A (Solaris)	Reverse primer	GACTCAGGTTCTCACGAATC
HIF2A (Solaris)	Probe	GCGACCATGAGGAGATT
VEGF (Solaris)	Forward primer	CATCACCATGCAGATTATGCG
VEGF (Solaris)	Reverse primer	GCTGTAGGAAGCTCATCTC
VEGF (Solaris)	Probe	CAAGGCCAGCACATAGGAG
PHD2 (Solaris)	Forward primer	AGCCCAGTTTGCTGACATTG
PHD2 (Solaris)	Reverse primer	CCAAACAGTTATTGCGT
PHD2 (Solaris)	Probe	TATGCTACAAGGTACGC
ERG (Solaris)	Forward primer	ACACCGTTGGGATGAACT
ERG (Solaris)	Reverse primer	TACTCCATAGCGTAGGATCTGC
ERG (Solaris)	Probe	AGAGTTATCGTGCCAGC
CTNNB1 (Solaris)	Forward primer	TGGCTATTACGACAGACTGC
CTNNB1 (Solaris)	Reverse primer	AGCCAGTATGATGAGCTTGC
CTNNB1 (Solaris)	Probe	TTATGGCAACCAAGAAAGC

© 2013 The Author(s)
The author(s) has paid for this article to be freely available under the terms of the Creative Commons Attribution Licence (CC-BY) (http://creativecommons.org/licenses/by/3.0/) which permits unrestricted use, distribution and reproduction in any medium, provided the original work is properly cited.

Table S3 Sequences of FISH probes

DNA probe	Sequence (5′→3′)
HIF1A sense control 1	CTCACAGATGATGGTGACATGATTTACATTTCTGATAATGTGAACAAATACATGGGATTA
HIF1A sense control 2	ATGGATGATGACTTCCAGTTACGTTCCTTCGATCAGTTGTCACCATTAGAAAGCAGTTCC
HIF1A sense control 3	CTATGTAGTTGTGGAAGTTTATGCTAATATTGTGTAACTGATATTAAACCTAAATGTTCT
HIF1A probe (antisense) 1	GAGTGTCTACTACCACTGTACTAAATGTAAAGACTATTACACTTGTTTATGTACCCTAAT
HIF1A probe (antisense) 2	TACCTACTACTGAAGGTCAATGCAAGGAAGCTAGTCAACAGTGGTAATCTTTCGTCAAGG
HIF1A probe (antisense) 3	GATACATCAACACCTTCAAATACGATTATAACACATTGACTATAATTTGGATTTACAAGA

Table S4 List of USP52-interacting proteins identified from MS/MS analysis

Available as an Excel file at http://www.biochemj.org/bj/451/bj4510185add.htm

Received 4 January 2013/31 January 2013; accepted 11 February 2013
Published as BJ Immediate Publication 11 February 2013, doi:10.1042/BJ20130026

© 2013 The Author(s)
The author(s) has paid for this article to be freely available under the terms of the Creative Commons Attribution Licence (CC-BY) (http://creativecommons.org/licenses/by/3.0/) which permits unrestricted use, distribution and reproduction in any medium, provided the original work is properly cited.
